# Mr. George William Powell

**Published:** 1911-04-01

**Authors:** 


At the early age of forty-nine Mr. George William Powell, B.A., M.B., Physician to the Birmingham Children's Hospital, has died at Edgbaston. He studied at Vienna and at Dublin, of which he was B.A. and M.B., B.C. He had held other appointments at the Children's Hospital, including that of Resident Medical Officer.

